# A Platform (Authorships.org) for the Objective Qualification and Order of Academic Authorship in Medical and Science Journals: Development and Evaluation Study Using the Design Science Research Methodology

**DOI:** 10.2196/34258

**Published:** 2022-03-17

**Authors:** Dimitri Aristotle Raptis, Aristotle Raptis, Pascale Tinguely, Authorships.org Collaborative

**Affiliations:** 1 Department of Hepato-Pancreatico-Biliary Surgery and Liver Transplantation Royal Free Hospital London United Kingdom; 2 Division of Surgical and Interventional Science University College London London United Kingdom; 3 Department of Surgery and Transplantation University Hospital Zurich Zurich Switzerland; 4 Division of Information and Communications Technology University of Athens Athens Greece; 5 See Acknowledgments

**Keywords:** authorship, writing, dissent and disputes, research ethics, software design

## Abstract

**Background:**

The qualification and order of authorship in scientific manuscripts are the main disputes in collaborative research work.

**Objective:**

The aim of this project was to develop an open-access web-based platform for objective decision-making of authorship qualification and order in medical and science journals.

**Methods:**

The design science process methodology was used to develop suitable software for authorship qualification and order. The first part of the software was designed to differentiate between qualification for authorship versus acknowledgment, using items of the recommendations of the International Committee of Medical Journal Editors. The second part addressed the order of authorship, using the analytical hierarchy process for objective multiple criteria decision-making and ranking. The platform was evaluated qualitatively (n=30) and quantitatively (n=18) using a dedicated questionnaire, by an international panel of medical and biomedical professionals and research collaborators worldwide.

**Results:**

Authorships.org represents an open-access software compatible with all major platforms and web browsers. Software usability and output were evaluated and presented for 3 existing clinical and biomedical research studies. All 18 international evaluators felt that the Authorships.org platform was easy to use or remained neutral. Moreover, 59% (n=10) were satisfied with the software output results while the rest were unsure, 59% (n=10) would definitely use it for future projects while 41% (n=7) would consider it, 94% (n=16) felt it may prove useful to eliminate disputes regarding authorship, 82% (n=14) felt that it should become mandatory for manuscript submission to journals, and 53% (n=9) raised concerns regarding the potential unethical use of the software as a tool.

**Conclusions:**

Authorships.org allows transparent evaluation of authorship qualification and order in academic medical and science journals. Objectified proof of authorship contributions may become mandatory during manuscript submission in high-quality academic journals.

## Introduction

Next to the presentation of scientific results, authorship in academic journal articles is a means for scholars to communicate the intellectual contributions of their work, take public responsibility for it, build reputation among peers, and convey their professional benefits [1]. However, scholars frequently encounter disputes concerning authorship, with the qualification and order of authorship remaining the main controversial issues in most collaborative work worldwide [2]. In other words, most dissents involve “Who are the authors, who should be acknowledged, and what should the order of the authors be in a given manuscript?” Numerous types of authorship abuses are considered scientific misconduct, with coercion (ie, intimidation tactics to gain authorship), guest, mutual support, ghost, and denial of authorship being the most frequent ones [3].

To address these issues, the International Committee of Medical Journal Editors (ICMJE) [4] established authorship guidelines including items related to study conception, execution, and documentation, which are adopted by many prominent institutions and journals worldwide [5]. While providing specific definitions regarding the roles of individual authors, the ICMJE guidelines do not allow a quantitative objectifiable assessment of individual author contributions within a body of authors, and disputes regarding authorship and the order of appearance frequently remain [6,7].

With the interest of promoting the highest ethics in medical and science publications, the aim of this project was to develop a user-friendly, open access, web-based software platform for the objective assessment of authorship qualification and order, in an attempt to reduce and hopefully eliminate authorship disputes.

## Methods

### Study Design

The design science research methodology was used to develop a suitable software for achieving our objective, as previously described [8]. Briefly, this is an established set of analytical techniques for performing research in information systems, involving the design of innovative products. The design science research methodology typically involves the problem identification, solution design, and evaluation phases [9].

### Settings, Developers, and Collaborators

The software was designed by DAR located in Switzerland and developed together with AR located in Athens, Greece, starting on May 7, 2016. A group of collaborators worldwide contributed in the design, development, and evaluation of the software, and were selected based on personal contacts and through personal interest, with a full list of names available in the Acknowledgments and on Authorships.org. A subset of 18 of these collaborators were selected for formal software evaluation as described below, with eligibility for selection being a scientific background in medicine or other biomedical areas, regardless of the grade. The characteristics of the collaborators who formally evaluated the software can be found in Table 1. The overall software design and development duration was from May 2016 to December 2019. The software evaluation took place between March and October 2016, with the headquarters of software evaluation located in Zurich, Switzerland.

**Table 1:** Characteristics of the collaborators formally evaluating the software.

Gender	Job title	Academic degree	Speciality	Country
Male	Attending	MSc, MD, PhD	Surgery	Switzerland
Male	Research scientist	MD, PhD	Internal medicine	United States
Male	Assistant professor	BSc, PhD	Information technology	Switzerland
Male	Resident	MD	Surgery	Switzerland
Female	Resident	MD	Surgery	United Kingdom
Male	Resident	MD	Surgery	Switzerland
Male	Research scientist	BSc	Information technology	Switzerland
Male	Attending	MD	Surgery	Switzerland
Female	Resident	MD	Internal medicine	Switzerland
Male	Attending	MD	Internal medicine	Germany
Male	Attending	MD	Surgery	Switzerland
Male	Research scientist	MD	Surgery	Switzerland
Male	Attending	MD	Surgery	Switzerland
Male	Research scientist	MD	Basic science	Australia
Female	Resident	MD	Surgery	Switzerland
Male	Resident	BSc, Dipl Eng, MD	Internal medicine	Switzerland
Male	Resident	MD	Internal medicine	Switzerland
Male	Attending	MD	Surgery	Switzerland

### Software Design and Development

The free web-based software is built on Drupal version 7 [10], an open-source content management system written in PHP and distributed under the GNU General Public License [11], as previously described [8]. An Apache server with a MySQL database was used [12]. The most advanced firewalls were installed for monitoring and prevention of malware. The costs for the server rental, domain name purchase, and software development were set at 2160 CHF (US $2332). These costs were successfully covered through a Kickstarter campaign by the collaborators on April 4, 2016 [13]. The website and software compatibility for different platforms, internet browsers, and devices were assessed using BrowserStack [14].

**Figure 1:**
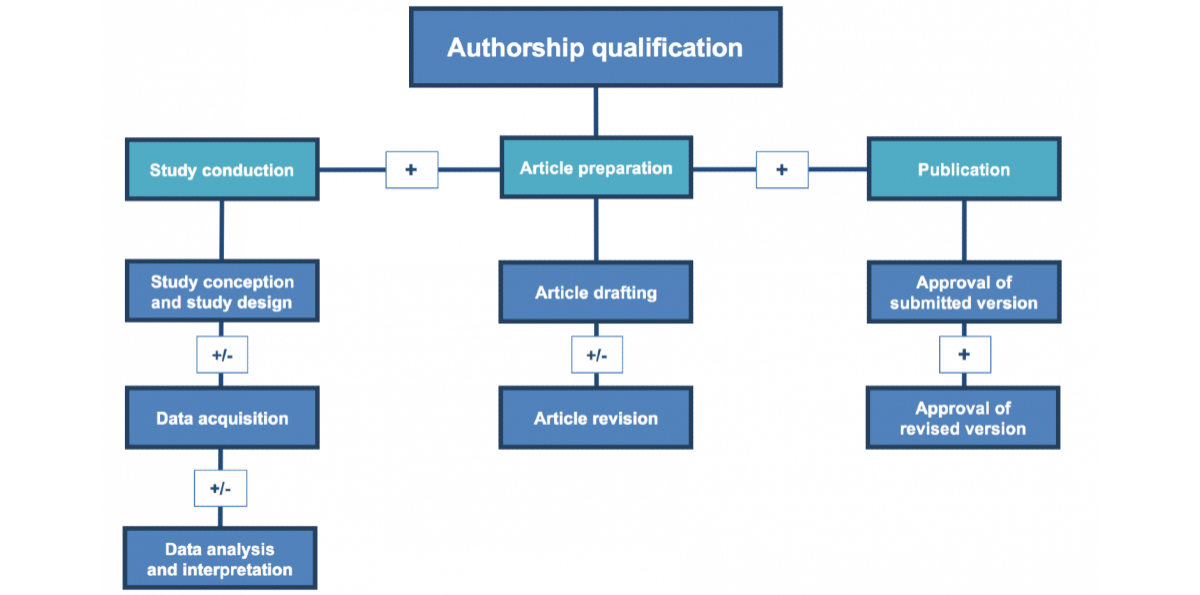
Qualification for authorship versus acknowledgment.

The first part of the software design focused on identifying and differentiating individuals whose work qualifies them as authors as opposed to contributors for acknowledgment. Items of the International Committee of “Medical Journal Editors Uniform Requirements for Manuscripts Submitted to Biomedical Journals,” including conception, execution, and documentation of research, were used to design the first part of the software (Figure 1) [4].

**Figure 2:**
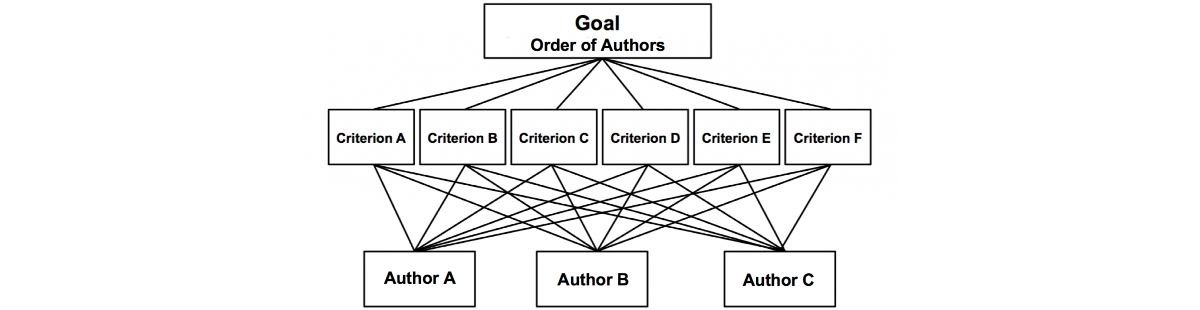
Analytical hierarchy process method for multiple comparisons and ranking of authors’ contributions.

The second part was designed to address the order of the qualifying authors using the analytical hierarchy process (AHP) method for objective multiple criteria decision-making and ranking [15]. Briefly, the AHP first decomposes the decision-making problem into a hierarchy of subproblems. Then, the relative weight of importance of the different criteria is assessed by pairwise comparisons. These weights are then used to calculate a score for each selection alternative. Information is decomposed into a hierarchy of alternatives, and criteria information is then synthesized to determine the relative ranking of alternatives. Both qualitative and quantitative information can be compared using informed judgements to derive weights and priorities. The consistency index measures the extent to which the decision-makers were consistent in their responses (Figure 2) [16].

### Software Evaluation

The software was evaluated and re-evaluated during the design phase as described above. At the end of software development, 18 collaborators (Table 1) assessed software usability and output in 3 existing clinical and biomedical research projects. The collaborators were chosen from the international panel of medical and biomedical professionals, based on the availability of a current research manuscript ready for submission and willingness to apply Authorships.org to evaluate their choice and order of authors. Collaborators were further asked to complete an online questionnaire (Multimedia Appendix 1). The evaluation questionnaire was designed by the authors, focusing on assessing the collaborator’s attributes regarding authorship disputes, software functionality and usability, and the need for improvements [17]. Descriptive statistics were used to report results using rBiostatistics [18].

## Results

### Software Design

The developed software, named Authorships.org [19], is freely available online and is compatible with all major platforms, web browsers, and mobile or tablet devices [14]. Authorships.org performs server-side calculations and graphical rendering, which eliminates any hardware requirements or incompatibilities at the user side.

**Figure 3:**
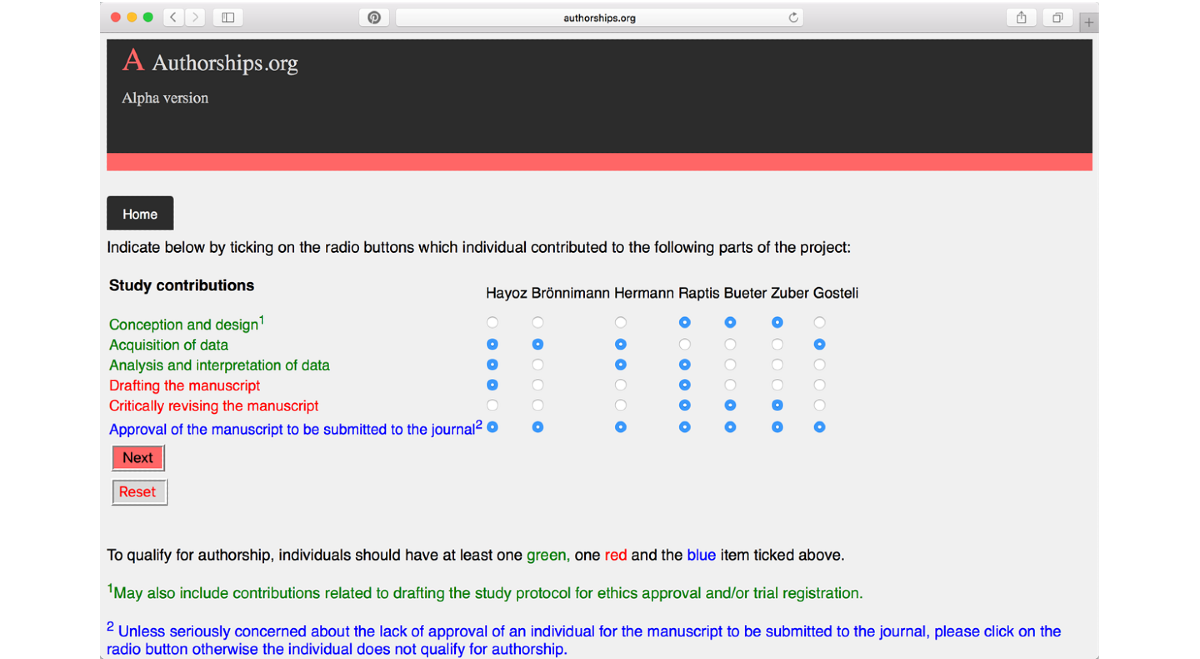
Selection of study contributions according to the International Committee of Medical Journal Editors (ICMJE) criteria [4].

First, the user is requested to add the number and names of the individuals who participated in a particular research project. Next, their contribution to the work is assessed based on the generally agreed upon ICMJE criteria, such as conception, execution, documentation, and final approval for publication (Figure 3) [4]. The user is also given the choice to give equal or different weights to these criteria, in order to respect local norms at different institutions worldwide.

**Figure 4:**
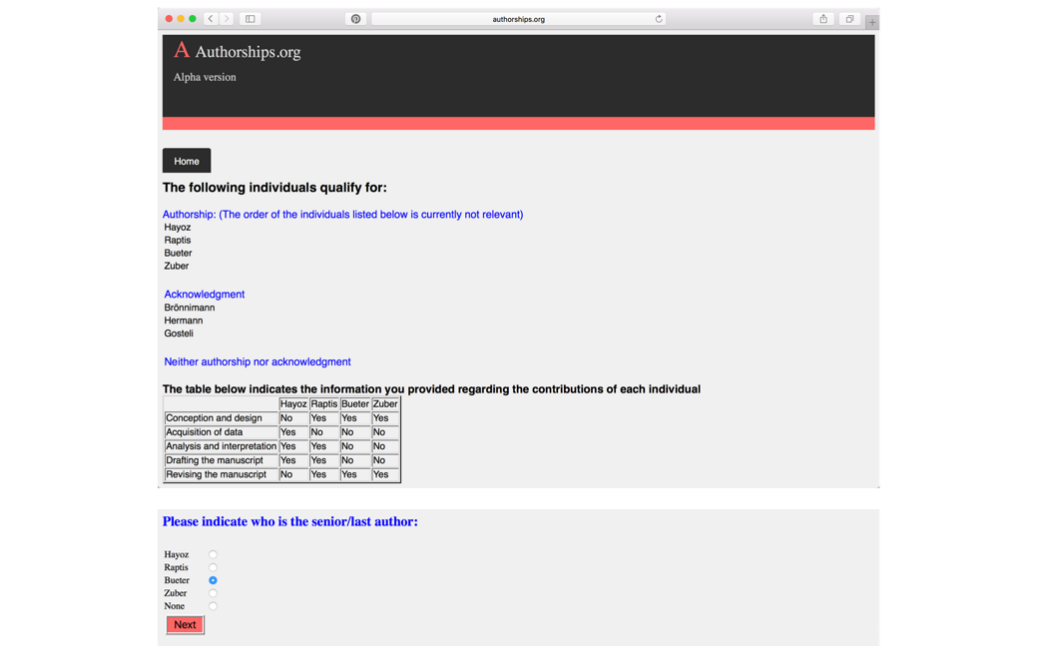
Software output on qualification for authorship versus acknowledgement.

Based on the existing ICMJE guidelines, the software then indicates which individuals qualify for formal authorship versus for acknowledgment as a contributor. The user is subsequently asked to indicate a senior/last author of the manuscript, if required (Figure 4).

**Figure 5:**
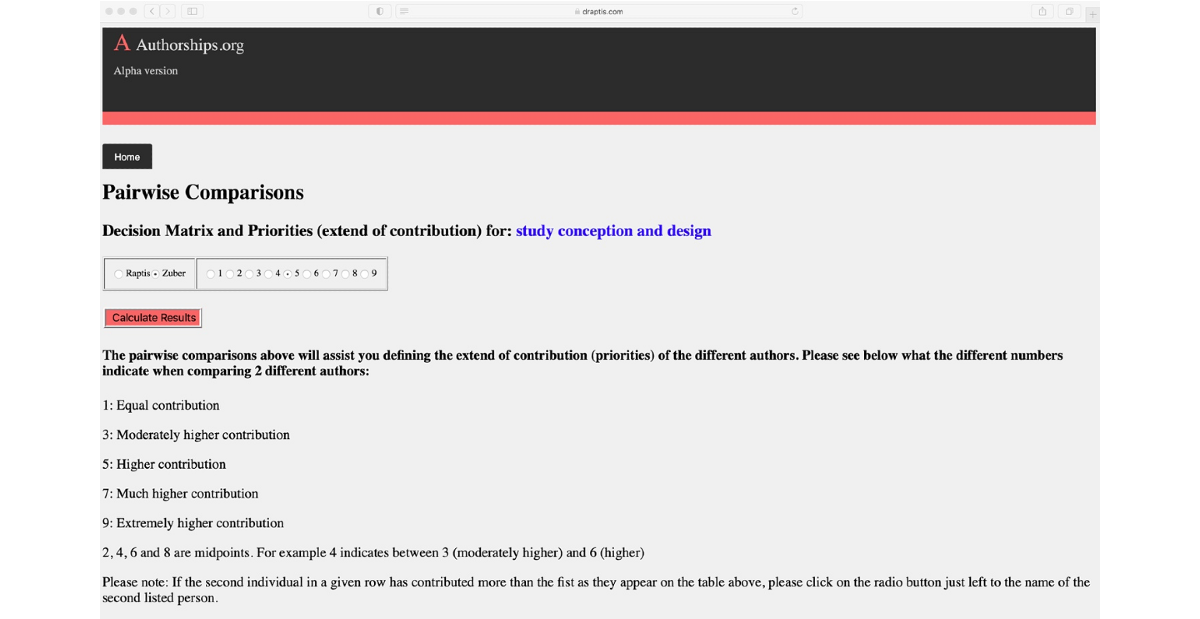
Example of pairwise comparison of the extent of individual author contributions for the International Committee of Medical Journal Editors (ICMJE) criterion of “study concept and design”.

In the second part, the user assesses the extent of the individual contributions of all qualifying authors, except the one defined as senior/last author. A ranking of each author’s extent of contribution to each ICMJE criterion is performed by using the AHP multiple comparisons method. Thereby, each author is compared to each other author in pairwise comparisons, using a decision matrix for ranking of the extent of contribution (Figure 5).

**Figure 6:**
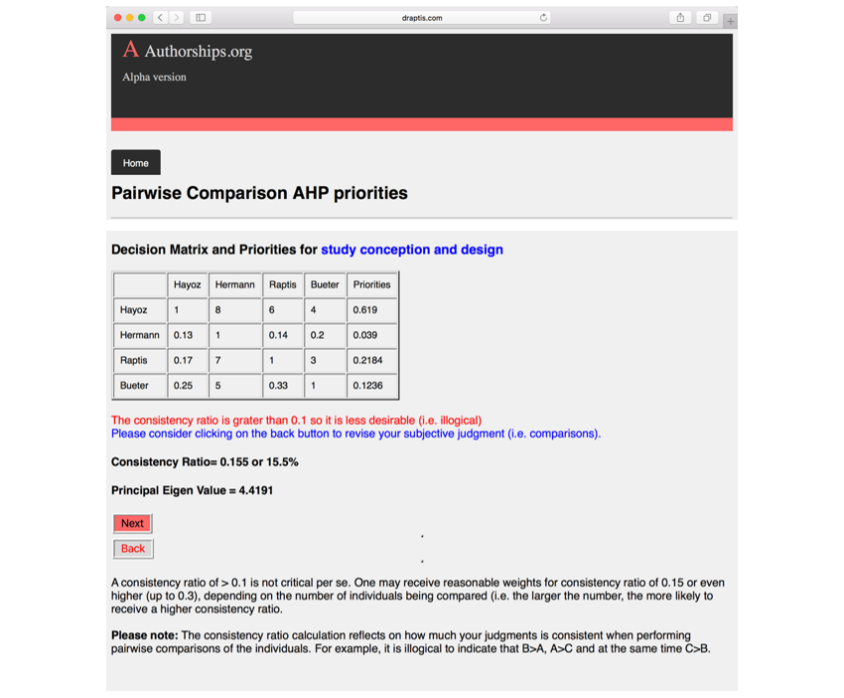
Software output on pairwise author comparisons and evaluation consistency.

After all pairwise comparisons are made, a consistency ratio is provided to the user to ensure the input data are consistent and logical (Figure 6). This ratio should typically be below 0.10; however, values as high as 0.30 are acceptable, especially when large numbers of authors and their contributions are being assessed [15]. In case of severe inconsistency, the user is required to repeat the evaluation steps of the second part, ensuring comparisons are meaningful (eg, A > B, B > C, A > C).

**Figure 7:**
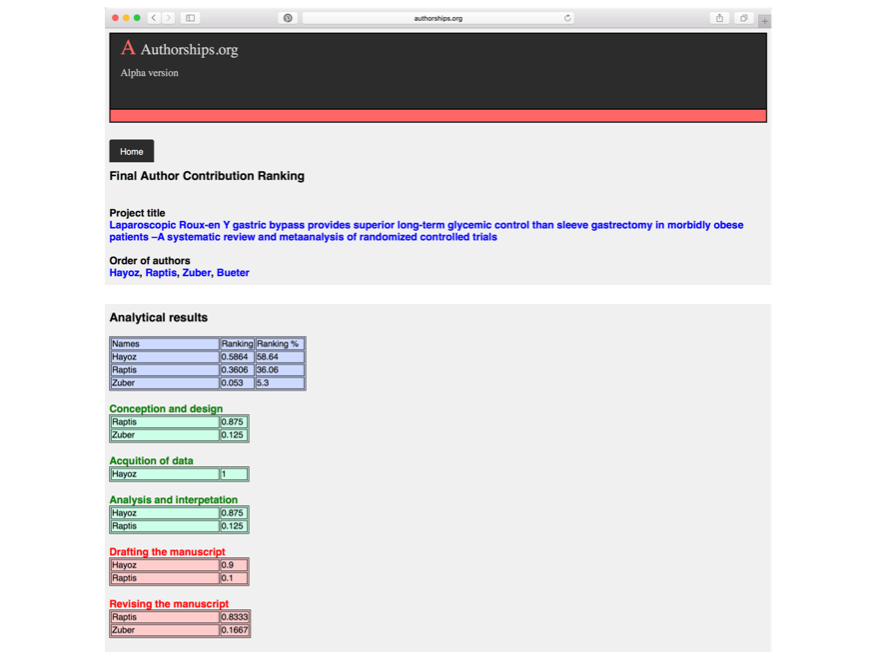
Software output on final author contribution ranking.

Based on these quantitative and objective evaluations, the software defines the appropriate order of author appearance in a publication at a medical or science journal (Figure 7).

### Software Evaluation

#### Questionnaire

**Figure 8:**
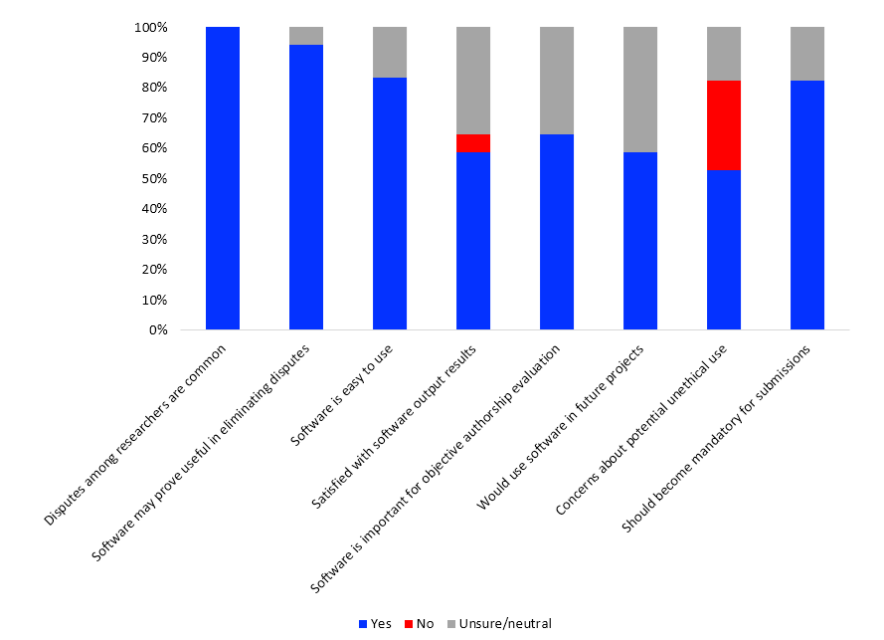
Results from the software evaluation questionnaire survey.

Eighteen international collaborators evaluated the software using the specially designed questionnaire. Of these, 88% (n=15) felt that the software was easy to use, while 12% (n=3) remained neutral. Additionally, 59% (n=10) were satisfied with the software output results, feeling that it objectively reflects reality, while 35% (n=6) were unsure and 6% (n=1) were dissatisfied. The reason stated for dissatisfaction was that the collaborator felt “one very important contribution (ie, outstanding critical manuscript revision) may be sufficient to qualify for authorship;” however, authors with such contributions were disqualified when using Authorships.org. Fifty-nine percent (n=10) stated they would definitively use the software in future projects, while 41% (n=7) would potentially consider it. Moreover, 94% (n=16) felt that it may prove useful to eliminate disputes regarding authorship, and 82% (n=14) felt that it should become mandatory for manuscript submission to journals. Fifty-three percent (n=9) raised concerns regarding potential unethical use of the software as a tool for authorship evaluation. Reasons given were concerns that Authorships.org may represent one more tool to “justify” unethical authorship behavior. The quantitative results are summarized in Figure 8.

#### Case Study 1

In an attempt to objectify authorship disputes, 6 collaborators contributing to a clinical study were asked to provide the authors’ order and reasoning behind it for a manuscript not yet submitted. The results of this survey were analyzed in a particular form developed at Authorships.org. Although they all agreed 100% regarding the authorship qualification of all 6 collaborators, a 0% total agreement on the order of author appearance was noted (Fleiss kappa: −0.10, 95% CI −0.18 to −0.01). In retrospect, the authors felt that the main reasons for this discordance were misconceptions on the amount of work conducted by their colleagues, lack of adherence to the “International Committee of Medical Journal Editors” recommendations, and some form of bias. Authorships.org was then applied to review the authorship order, yielding complete agreement of the collaborators. The collaborators felt that the platform was a transparent and objective tool for assigning authorship order, and justified the use of Authorships.org for their work.

#### Case Study 2

Seven authors of a biomedical engineering manuscript based on clinical and medical imaging data were asked to provide the order of the authors for the paper they all contributed. All authors agreed on their own qualification for authorship, and 2 (28%) authors agreed about the collaborator order for the manuscript. Authorships.org was used to assess author qualification and the order of authorship. Thereafter, 5 out of the 7 authors justified the use of Authorships.org and thought that it is a robust and objective tool to assign the order of authors. The reason for the disagreement of the remaining 2 authors was a feeling that Authorships.org did not sufficiently acknowledge the seniority level of individual authors. They further disagreed to the design that authors should fulfil at least one green item, one red item, and the blue item to qualify for authorship in Autorship.org (based on the ICMJE recommendations) (Figure 3).

#### Case Study 3

Eight members of an international research group conducted a systematic review and meta-analysis regarding the use of comprehensive enhanced recovery protocols in the setting of liver transplantation. Individual tasks included study design and methodology, screening of titles and abstracts of studies identified in the literature search, data extraction and analysis of selected works, manuscript writing, and critical review. Authorships.org was applied after drafting the manuscript and before submission to a peer-reviewed scientific journal. The objective author qualification and order were applied directly in the manuscript as suggested by the software output and accepted by all authors.

## Discussion

This study reports on the development and evaluation of a novel software tool for the objective assessment of authorship qualification and order, in an attempt to reduce authorship disputes. To the best of our knowledge, Authorships.org represents the first open-access web-based software to quantitatively and thus objectively indicate the qualification and order of academic authorship in medical and science journals.

The majority of collaborators assessing the usability of Authorships.org found the software helpful and easy to use, and felt that the software could have a high impact in the scientific community in a short period of time. The concerns of the remaining collaborators regarding software output results focused on the criteria required for authorship qualification and therefore on the internationally accepted ICMJE criteria applied in the proposed software algorithm. While the qualification for authorship has been standardized in the ICMJE criteria [4], varying approaches of determining the order of authorship are currently applied across scientific disciplines, research groups, and countries. Examples of authorship policies include descending order of contribution, placing the person who took the lead in writing the manuscript or performing main research tasks first and the most experienced contributor last, or using an alphabetical or random order [1,20,21]. While the significance of a particular order may be understood in a given setting, the order of authorship is not generally agreed upon [22]. Attempts to reduce inaccuracies in author lists include models similar to the film credit concept, with mandatory contribution statements replacing the author’s list, such as Credit Taxonomy [23]. However, these could lead to a shift in importance from the authors who actually produced the science to a more confounding way of giving credit to each research co-operator. Autorships.org not only represents a quantifiable and thus objectifiable approach, but also could provide a solution for the correct interpretation of the respective contributions of individual authors. Since the indication of specific author contributions has become mandatory in most scientific journals with a mid to high impact factor [22], the submission of a separate document objectifying the assessment of author qualification and contributions, such as provided by Authorships.org, may become obligatory in the future. To this end, Authorships.org must be formally validated in future projects worldwide, including a randomized controlled study comparing the levels of disputes.

One main hypothetical advantage obtained by the implementation of an objective instrument in academic authorship, as presented herein, is a reduction in the number of disputes. Academic authorship of papers arising from complex research projects, involving multiple centers and with potential valuable impact on the scientific community, can nowadays be considered as a currency for career development. These scenarios represent feeding terrains for conflicts between authors, which can sometimes even result in the retraction of a manuscript from publication or, if remaining unresolved due to failed mediation among authors, in rejection by the journal. Another known issue is the abuse of power exerted by certain authors inflicting the order of authors in their own interest, which can be avoided by using an objectifiable tool such as Authorships.org. We believe that the most reasonable attempt to resolve such disputes is undertaken primarily among the authors themselves, and in case of failure to compromise, the disputes are taken up by the authors’ affiliating institutions. Moreover, Authorships.org allows authors to evaluate the extent of contribution provided by each author to individual phases of manuscript preparation, representing the main advantage of using the AHP over other ranking models. Another main advantage of the proposed algorithm is the possibility to “customize” the relevance of each area of performance according to the contributors’ previous agreement or the institution’s internal guidelines. The use of the software could prompt a prearranged settlement and, consequently, help the team working on the project on the basis of mutually approved rules (eg, first authorship for a PhD candidate or last authorship for professorship title). This further allows maintaining the “motivating” aspect of a researcher to perform at her/his highest level when aiming to keep a certain position within the authorship list. Most of the collaborators evaluating the software further felt that the added consistency ratio supported them in reflecting their own contributions to a project in a quantitative way. A progressive improvement in consistency ratio values with increasing use of the software is further expected, as a result of ongoing education on the use of a mathematical way to evaluate each participant’s contribution.

An important aspect of the Authorships.org design is the time point of the assessment of the qualification and order of authorship. While in many research groups worldwide, author roles are defined *prior to* the start of a research project, Authorships.org was deliberately designed to be applied *after* the execution and writing of a manuscript but before submission to a scientific journal, preferably in a discussion by all contributing members of the research team [1]. Hence, authorship qualification and order reflect the actual tasks performed by each individual rather than preset rigid orders potentially prone to abuse. It is essential to note that if not applied at the correct time point, Authorships.org may not be beneficial or, if abused, may even lead to erroneous results, which might represent a limitation to the current methodology. A further potential conflict of the proposed software is the option to change the weight of assessment for different parameters, which may increase the possibility of manipulation of weights toward personal interests. This was added to address the different needs across scientific fields. For example, in clinical research, more weight might be placed on the researcher who wrote the manuscript, whereas in basic science, more weight might be placed on the researcher performing the laboratory work. In general, we recommend giving equal weight to all assessment parameters. The acknowledgment of the seniority level of individual authors has been raised as a critical point by several collaborators evaluating Authorships.org. The software was designed such that 1 senior/last author could be specifically chosen based on her/his a priori qualification as the senior project leader. This was deliberately limited to 1 person to avoid multiple senior authors being listed for “political” reasons, acknowledging internal hierarchical structures rather actual contributions to a scientific work. While abusive authorship selections may still remain when using Authorships.org, the use of the proposed software has the potential to make such conflicts more transparent, raise awareness among authors, and therefore contribute to open discussions about conflicts in authorship contributions. Finally, views of the authorship disputes and attributes, as evaluated by the collaborators, might not reflect generalizable views across researchers. However, with the selected collaborators originating from countries across the world and from varying areas of science, this potential selection bias might have been alleviated.

In conclusion, Authorships.org represents a novel approach to quantify and objectify the qualification and order of authorship in academic literature. It may become a mandatory tool for objectified proof of author contributions in scientific publications. Further randomized studies are needed to validate the potential of using such a tool for reducing or eliminating authorship disputes.

## Appendix

Multimedia Appendix 1 Online questionnaire.

## Abbreviations

AHP: analytical hierarchy process
ICMJE: International Committee of Medical Journal Editors
