# Supplementary material for: A Platform (Authorships.org) for the Objective Qualification and Order of Academic Authorship in Medical and Science Journals: Development and Evaluation Study Using the Design Science Research Methodology
Source: JMIR Form Res. 2022 Mar 17;6(3):e34258. doi: 10.2196/34258 (PMC8972106; doi:10.2196/34258)
Supplement: Multimedia Appendix 1 [file formative_v6i3e34258_app1.docx]

Authorships.org Evaluation

How did you hear about Authorships.org? ___________________________________________

Disputes among researchers regarding authorship qualification and order are quite common: *

〇 Strongly disagree

〇 Disagree

〇 Somewhat disagree

〇 Neither agree or disagree

〇 Somewhat agree

〇 Agree

〇 Strongly agree

Authorships.org is an easy to use software *

〇 Very difficult

〇 Difficult

〇 Neutral

〇 Easy

〇 Very easy

Authorships.org software needs improvements *

〇 Strongly disagree

〇 Disagree

〇 Somewhat disagree

〇 Neither agree or disagree

〇 Somewhat agree

〇 Agree

〇 Strongly agree

If you agree, please explain which improvements _____________________________________

Would you consider using Authorships.org in every project and manuscript you are involved in? *

〇 Would definitely not consider

〇 Might or might not consider

〇 Would definitely consider

Authorships.org software has problems and bugs *

〇 Strongly disagree

〇 Disagree

〇 Somewhat disagree

〇 Neither agree or disagree

〇 Somewhat agree

〇 Agree

〇 Strongly agree

If you agree, please explain the problems ___________________________________________

Authorships.org is important for objective qualification and order of authorship *

〇 Not at all important

〇 Low importance

〇 Slightly important

〇 Neutral

〇 Moderately important

〇 Very important

〇 Extremely important

Authorships.org is useful to decrease or eliminate disputes regarding authorship *

〇 Strongly disagree

〇 Disagree

〇 Somewhat disagree

〇 Neither agree or disagree

〇 Somewhat agree

〇 Agree

〇 Strongly agree

Are you satisfied with the Authorships.org output results of the author qualification and ranking? Do they objectively reflect reality? *

〇 Very dissatisfied

〇 Dissatisfied

〇 Unsure

〇 Satisfied

〇 Very satisfied

If dissatisfied, please explain why _________________________________________________

Authorships.org may be still used unethically as a tool *

〇 Not at all concerned

〇 Slightly concerned

〇 Somewhat concerned

〇 Moderately concerned

〇 Extremely concerned

If concerned, please explain why ________________________________________________

It should become mandatory to submit to a journal the Authorships.org output results of the author qualification and ranking along with a manuscript *

〇 Strongly disagree

〇 Disagree

〇 Somewhat disagree

〇 Neither agree or disagree

〇 Somewhat agree

〇 Agree

〇 Strongly agree

General remarks (optional) ______________________________________________________
